# Direct observation of outpatient management of malaria in a rural ghanaian district

**DOI:** 10.11604/pamj.2014.19.367.4719

**Published:** 2014-12-10

**Authors:** Donne Kofi Ameme, Edwin Andrews Afari, Kofi Mensah Nyarko, Keziah Laurencia Malm, Samuel Sackey, Fred Wurapa

**Affiliations:** 1 Ghana Field Epidemiology and Laboratory Training Programme (GFELTP), School of Public Health, University of Ghana, Accra, Ghana; 2 Disease Control and Prevention Department, Ghana Health Service, Accra, Ghana

**Keywords:** Malaria, case-management, Ghana, observation

## Abstract

**Introduction:**

In Ghana, malaria continues to top outpatient morbidities; accounting for about 40% of all attendances. Effective case-management is key to its control. We evaluated case-management practices of uncomplicated malaria in Kwahu South District (KSD) health facilities to determine their conformity to guidelines.

**Methods:**

We conducted a cross sectional survey at all public health facilities in three randomly selected sub-districts in KSD. A non-participatory observation of suspected malaria consultations was conducted. Suspected malaria was defined as any person with fever (by history or measured axillary temperature > or equal 37.5 oC) presenting at the selected health facilities between 19th and 29th April 2013. Findings were expressed as frequencies, relative frequencies, mean (± standard deviation) and median.

**Results:**

Of 70 clinical observations involving 10 prescribers in six health facilities, 40 (57.1%) were females and 16 (22.9%) were below five years. Median age was 18 years (interquartile range: 5-33). Overall, 63 (90.0%) suspected case-patients had diagnostic tests. Two (3.6%) were treated presumptively. All 31 confirmed and 10 (33.3%) of the test negative case-patients received Artemisinin-based Combination Therapies (ACTs). However, only 12 (27.9%) of the 43 case-patients treated with ACT received Artesunate-Amodiaquine (AA). Only three (18.8%) of the under-fives were examined for non-malarial causes of fever. Mean number of drugs per patient was 3.7 drugs (± 1.1). Only 45 (64.3%) patients received at least one counseling message.

**Conclusion:**

Conformity of malaria case-management practices to guidelines in KSD was suboptimal. Apart from high rate of diagnostic testing and ACT use, prescription of AA, physical examination and counseling needed improvement.

## Introduction

Despite remarkable progress in malaria control in sub-Saharan Africa [1], the disease remains a major public health problem with its effects amplified in children and pregnant women in developing countries [2]. Though preventable and curable, malaria causes high morbidity and mortality. The majority of this mortality burden is borne by sub-Saharan Africa where an estimated one million deaths occur with three fourth of these deaths occurring in children under five years. This translates to one child in Africa dying from malaria every minute [2]. In Ghana, malaria ranks first among outpatient diagnoses, accounting for approximately 40% of all out-patient department diagnoses [3]. Effective case-management, however, has been recognized as a fundamental pillar in malaria control [4–6]. It reduces morbidity and mortality as well as contributes to reduction in the transmission of the disease [2]. The World Health Organization (WHO) recommends diagnostic testing of all suspected malaria cases and targeted treatment of confirmed uncomplicated malaria with Artemisinin-based Combination Therapy (ACT) [2, 7]. ACTs have been widely accepted as first line treatment of uncomplicated malaria in Africa [8]. Uncomplicated malaria refers to malaria with non-specific symptoms such as fever, chills, joint pains and headache without features characteristic of severe disease such as blood or metabolic abnormalities and serious organ dysfunction.

Ghana adopted the use of highly effective ACTs in 2004 for treatment of uncomplicated malaria [9] following widespread parasite resistance to chloroquinemonotherapy [10]. According to Ghana's current antimalarial policy, Artesunate-Amodiaquine (AA) is the first line drug for uncomplicated malaria with Artemether-Lumefantrine (AL) and Dihydroartemisinin-Pyperaquine as alternatives for those who cannot tolerate AA [11]. There has been continuous sensitization of health workers on the case-management guidelines developed by the Ministry of Health [3]. However, conformity of health workers management practices to these guidelines remains doubtful. High reported malaria caseloads have been blamed on rampant presumptive diagnosis by health workers [3, 12]. Non-conformity of health workers to guidelines had also been previously documented in Ghana [13]. However, assessment of the current malaria case-management practices is central to the development of targeted malaria control interventions. Malaria case-management practices have been assessed using different approaches [13, 14]. However, this subject has not been explored much in Ghana using direct observation of the health worker-patient consultations. We therefore evaluated health worker malaria case-management practices by observing outpatient consultations in the Kwahu South District (KSD) of the Eastern Region of Ghana as a basis for health worker focused interventions.

## Methods

**Study design**: we conducted a health facility based cross sectional survey in public health facilities in KSD from 19th April to 29th April 2013. Direct observation of the health worker-patient interactions and interviews were used to assess case- management practices.

**Figure 1:**
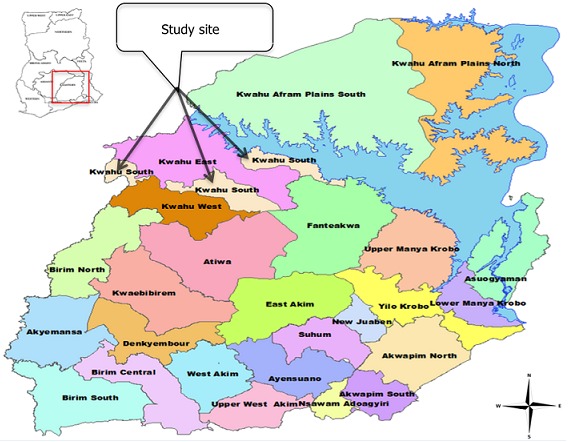
Map of Eastern Region of Ghana showing the 26 districts including Kwahu South District. The insert depicts the location of the Eastern Region in Ghana

**Study area**: KSD is one of the 26 districts in the Eastern Region of Ghana (Figure 1) with a total population of 69, 757 [15]; 28% of which represents the population of children under five years of age. The district capital, Mpraeso, is about 200km from Accra, the capital city of Ghana. KSD is a predominantly rural district with a rural urban ratio of 1: 0.4. The district has a male population of 33,094 giving a sex ratio of 1: 1.1 [15]. There are 17 public health facilities including the district hospital serving all six sub-districts. Health care delivery is organized at different levels from the lowest level of care known as the Community-based Health Planning and Services (CHPS) compounds, which are manned by community health nurses. The prescribers at the health centres are higher cadre nurses as well as physician assistants. The district hospital has physician assistants and medical doctors as prescribers. Malaria transmission in the district is endemic with seasonal fluctuations. Higher malaria transmission occurs during the major rainy season between April and July annually.

**Sampling Procedure**: fifty percent of the sub-districts were randomly selected for the study. Each of the sub-districts has at least one health facility. All of the public health facilities in the selected sub-districts were included in the study. All health workers who were performing outpatient consultations in the selected health facilities during the study period were included. We selected patients for observation using an adaptation of the Lots Quality Assurance Sampling (LQAS) technique for health worker skill detection [16]. This is based on the assumption that if health workers are expected to deliver services using the correct technique at least 95% of the time; then using the binomial equation, at least six observations per health worker are needed to determine this skill level [16]. Six clinical observations per health facility had also been used by Schellenberg et al [17, 18] though another study involving observation of health worker-patient interaction did two observations per condition in each facility [19]. Some other studies did observation for a certain time period ranging from two days [20] to two weeks [21]. We observed seven outpatient consultations per health worker making allowance for one observation that may not have been done well.

**Data collection technique, tools and procedure**: all the seven public health facilities from the selected sub-districts were visited. However, one CHPS compound, located in the Mpraeso sub-district was excluded from the study on account of small caseload of less than one patient per day. This facility had temporarily stopped attending to patients on the National Health Insurance Scheme (NHIS) as a result of unresolved administrative issues. The trained survey team visited the remaining six health facilities early in the mornings of selected days of the week. The team introduced its members to the authorities of the health facilities and sought permission for the commencement of the study. At each health facility, a primary sampling unit for observation was a health worker-patient consultation. All health workers performing general outpatient consultation were eligible for inclusion and agreed to be included. A suspected malaria case-patient was any patient aged 2 months and above, presenting for an initial visit with fever (by history or measured axillary temperature of > or equal 37.5 oC) to any of the selected health facilities. Any patient who met these criteria was eligible for inclusion in the study. Patients less than two months old, pregnant women, seriously ill patients, patients attending the health facility for follow up, patients whose presenting complaints qualified them for specialized care and patients attending the health facility for chronic illnesses were excluded from the study. Patients who were coming for a follow-up on initial fever or malaria treatment were excluded to ensure the findings better represent initial malaria case-management practices. Patients less than two months old were also excluded because fever in patients less than two months old was less likely to be due to malaria. Patients arriving at the facility were recruited based on the inclusion criteria and their willingness to be part of the study. They were enrolled in the study for the observation process after seeking their informed consent. Consent was obtained on behalf of younger children from their caretakers. In addition to parental consent, assent was obtained from older children. The first seven sampling units that met the inclusion criteria were selected per each health worker at each health facility. This was based on the assumption that the pattern in which patients arrived at a health facility was not associated with quality of care they received [16]. Patients and caretakers who agreed to be part of the survey were recruited at the history table, given identification and their vital signs recorded before they entered the consulting rooms. During the consultation process, an independent observer observed and recorded all the assessment tasks performed, the diagnoses and the medications prescribed without participating in the interaction between the health worker and the patient. In cases where the observer was unable to read directly from the record, the health worker was asked about the diagnoses and the medications prescribed. This process was repeated for the other six patients per health worker recruited into the study. In the case of children, if more than one child per caretaker presented, one was selected at random for inclusion. The other children were excluded from the study and the next patient who met the inclusion criteria was sampled. All the prescribers attending to patients were included in the study, for observation of their consultations. After the observation, the health worker was interviewed either immediately or at the end of the entire consultation on their demographics, training, reasons for choice of anti-malarials and management tasks performed.

**Quality control**: the data collection tools were pre-tested in health facilities in another district, which has similar settings as the facilities in the KSD. This is to ensure the tools were well formatted and reflected the local conditions. The necessary modifications were made based on the pre-test. Two staff nurses were used as field workers in the data collection. They were trained and assessed to ensure they had the ability to accurately observe consultations and record study participants’ actions and responses. Simulated practices were repeated to increase the agreement and consistency between field workers and the trainer. During the data collection, the principal investigator supervised the field workers and data collected was randomly crosschecked for completeness. Data was double entered by two independent data entry clerks into Epi info software version 3.5.4. Discrepancies were resolved by referring to the original data collection tools.

**Data processing and analysis**: the primary outcome measure was appropriate management of malaria cases; defined as prescription of ACT for confirmed uncomplicated malaria and not prescribing ACT for test negative or presumptive malaria. We performed descriptive statistical analysis and expressed categorical variables as frequencies and relative frequencies. Continuous variables that were uniformly distributed were expressed as means (+/- standard deviation) and those non-uniformly distributed expressed as medians (interquartile ranges). In inferential statistical analysis, 95% CI was used to determine significant measures of effect. Data was entered, cleaned and analyzed using Epi info version 3.5.4 software.

**Ethical considerations**: ethical approval was obtained from the Ghana Health Service Ethical Review Committee and permission was sought from the Kwahu South District Health Directorate and the management of the selected health facilities prior to commencement of the study. Voluntary informed consent was obtained from the adult patients and caretakers of children. Assent was obtained from older children before participating in the study. Confidentiality was observed throughout the study.

## Results

**Health facility characteristics**: overall, six health facilities were included in the study. Of these, one (16.7%) was a hospital, three (50.0%) health centres, one (16.7%) clinic and two (33.3%) CHPS compounds. All the selected health facilities have diagnostic capacity during the study period. Blood film microscopy was the main diagnostic test available at the hospital whilst Rapid Diagnostic Testing (RDT) was the means of confirming malaria in the other health facilities.

**Health worker characteristics**: all the 10 health workers agreed to participate in the study and all were observed and interviewed. Two (20.0%) of the health workers were medical officers; three (30.0%) were medical assistants and the rest, various categories of nurses. Four (40.0%) were females. Two (20.0%) of the health workers did not have any formal training in malaria case-management and of the eight who received formal training, none was trained within six months preceding the survey period.

**Patient characteristics**: out of the 70 clinical observations, 40 (57.1%) were females and 16 (22.9%) were below five years (Table 1). The median age of the patients was 18 years (interquartile range; 5-33).

**Table 1:** Distribution of patients in clinical observation sample by age, sex, occupation and type of health facility, Kwahu South District, 2013

Patient Characteristics	Health Facility Type				Overall
	Hospital	Health Centre	Clinic	CHPS	
	n (%)	n (%)	n (%)	n (%)	n (%)
*Sex*					
Female	18 (51.4)	16 (76.2)	4 (51.7)	2 (28.6)	40 (57.1)
Male	17 (48.6)	5 (23.8)	3 (42.9)	5 (71.4)	30 (42.9)
*Age*					
0-4 years	3 (8.6)	7 (33.3)	4 (57.1)	2 (28.6)	16 (22.9)
5-12 years	7 (20.0)	2 (9.5)	0 (0.0)	4 (57.1)	13 (18.6)
>12 years	25 (71.4)	12 (57.1)	3 (42.9)	1 (14.3)	41 (58.6)
*Occupation*					
Farmer	6 (17.1)	5 (23.8)	2 (28.6)	1 (14.3)	14 (20.0)
Government employee	7 (20.0)	1 (4.8)	0 (0.0)	0 (0.0)	8 (11.4)
Trader	4 (11.4)	4 (19.0)	0 (0.0)	3 (42.9)	11 (15.7)
Unemployed	1 (2.9)	1 (4.8)	0 (0.0)	0 (0.0)	2 (2.9)
Pupil/ Student	12 (34.3)	3 (14.3)	1 (14.3)	1 (14.3)	17 (24.3)
Other	5 (14.3)	7 (33.3)	4 (57.1)	2 (28.6)	18 (25.7)
*Total*	35 (100.0)	21 (100.0)	7 (100.0)	7 (100.0)	70 (100.0)

**Figure 2:**
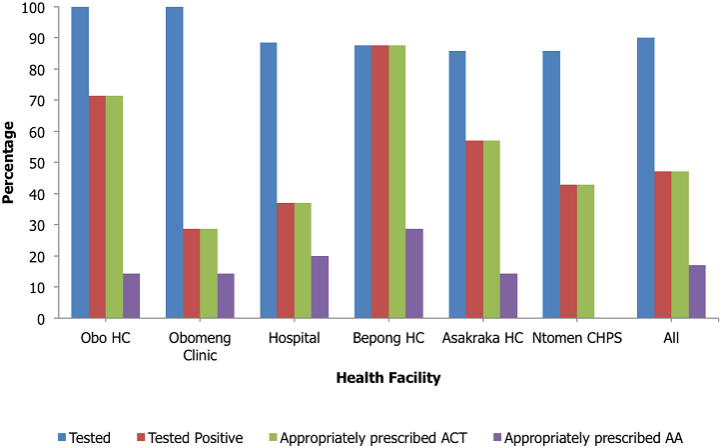
Proportion of suspected malaria cases tested (microscopy or RDT) and appropriately prescribed ACTs by health facility, Kwahu South District, 2013

**Figure 3:**
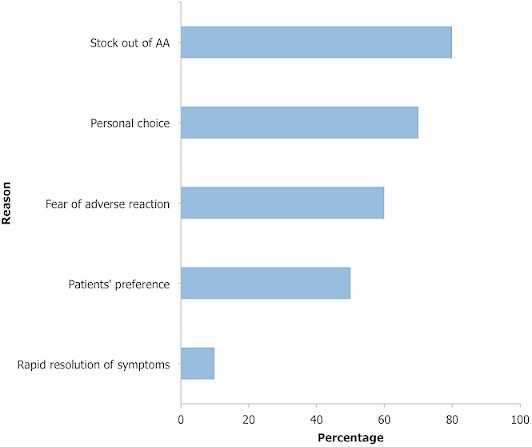
Health workers’ reasons for prescribing other anti-malarials other than AA, Kwahu South District, 2013

**Health worker prescription practices**: the proportion of suspected malaria cases tested and treated according to test results with ACT varied between facilities(Figure 2). Overall, as many as 63 (90.0%) of the patients were tested for malaria: 32 (50.8%) by RDT and 31 (49.2%) by blood film for malaria parasites. Of those who had diagnostic tests done, 33 (52.4%) tested positive for malaria and were appropriately treated with ACTs. However, only 12 (36.4%) of them received AA (Figure 2). The rest received alternate first line AL. In all, 45 (64.3%) patients including all 33 test positive, 10 test negative and 2 presumptively diagnosed malaria case-patients were treated for malaria with ACTs. Majority 33 (73.3%) of the 45 patients, who were treated with ACTs, received AL. The remaining 12 (26.7%) received AA. Overall, 53 (75.7%) patients comprising all 33 confirmed malaria case-patients and 20 (66.7%) of the test negative case-patients were treated according to test results. The mean number of drugs per patient was 3.6(±1.1). Only three (18.8%) of the patients under five years old had their ears, throat and chest examined for other non-malarial causes of fever. Majority (80%) of the health workers cited stock out of AA as the reason for prescribing other anti-malarials other than AA (Figure 3). Health workers personal choice and fear of adverse reactions were other reasons cited by 70% and 60% of the health workers respectively.

**Counseling of patients**: of the 10 health workers’ nine (90%) said they routinely counsel their patients. However, only 45 (64.3%) of the patients received at least one counseling message. The analysis included all the suspected malaria cases observed irrespective of their final diagnosis. All the observed patients had either a recent history of fever or measured fever and received at least one medication and were therefore included in the analysis. Each of the different categories of counseling messages was provided to less than a half of the patients (Table 2). The dosage regimen of the medications supplied was the most frequently provided counseling instruction observed in 24 (34.3%) cases. Telling patients and their caregivers the diagnoses was the next most frequently provided to 15 (21.4%) patients. Counseling on use of ITN, facility re-engagement instructions, instructions on home management of fever, and dietary advice followed in that order.

**Table 2:** Observed provision of counseling to suspected malaria patients by health workers, Kwahu South District, 2013

Counseling Instruction	N	Provided (%)	(95% CI)
Tell diagnosis	70	21.4	(12.5, 32.9)
Explain home management of fever	70	8.6	(3.2, 17.7)
Give dietary instructions	70	7.1	(2.4, 15.9)
ITN use	70	20.0	(11.4, 31.3)
Health facility re-engagement	70	8.6	(3.2, 17.7)
Dosage/ regimen of medications	70	34.3	(23.3, 46.6)

## Discussion

This evaluation assesses malaria case management practices in a rural setting in Ghana almost a decade after adoption of ACTs. Diagnostic testing of suspected malaria cases was almost universal (90%). A likely explanation for this high rate of diagnostic testing of suspected cases could be due to the fact that the prescribers were being observed. This high rate contrasts with findings of low rates of testing in other studies [22–25]. Testing is very critical in malaria case- management as it offers the opportunity to rule out other causes of fever thereby preventing over diagnosis and inappropriate use of ACTs. The high testing rate observed in this study therefore benefited the patients by providing the opportunity for excluding other causes of fever. Disregard for negative test results was quite appreciable (33.3%) but lower than 82% observed in Papua New Guinea [24] and 79.3% in Kenya [26]. The result of this, is inappropriate prescription of anti-malarials to test negative cases as evident in many studies [23–26]. Health workers lack of trust in the test results could be a likely explanation for this phenomenon as was observed in Angola [25]. This has implications on case-management. It prevents the evaluation of the patient for other non-malarial causes of fever. This means that, other potentially serious diseases that mimic malaria may be missed leading to over diagnosis and over treatment of malaria. With regards to prescription of ACT, all the patients who were diagnosed of malaria were treated with ACT in conformity to recommended guidelines. A similarly high proportion of patients prescribed ACT was observed by Sears et al [27]. Though all the patients were treated with ACT, use of AA was low in favour of alternative first line AL. The reason cited by most of the health workers for this phenomenon is stock out of AA, personal choice and fear of adverse effects. Widely reported adverse reactions that accompanied AA use [11] and the negative media reportage on its safety [14] when it was first introduced in Ghana could be lingering on. Assessment of the patients for other causes of fever was inadequate. Only a handful of children under five years were examined for other causes of fever. Incomplete assessment of children with suspected malaria has been documented in other studies [28, 29]. Since children under five years bear the brunt of malaria, it is expected that they be accorded more meticulous assessment in terms of history, examination and laboratory investigation. Gaps in any of these tasks may be detrimental to the children and malaria control in general.

Counseling of patients with suspected and confirmed malaria was highly inadequate. This finding compares well with an observation in Papua New Guinea [24] and Tanzania [30]. Of the six broad areas of counseling messages that we considered, none was given to at least half of the patients. This has far reaching consequences on case-management as it precludes full adherence of patients to treatment. Patients are therefore deprived of knowledge and insight into their condition and the need for follow up. The lack of recent training of the health workers in malaria case-management could explain the lapses observed. This study serves as a baseline of the case-management practices in the KSD. Regular health facility surveys on malaria case-management have been shown to significantly improve case-management indicators elsewhere and therefore recommended as a monitoring tool for African countries with needs in monitoring progress at subnational levels [31]. A rapid assessment method of this nature may be a useful initial step in realizing this recommendation. Our evaluation of the health workers malaria case-management reveals critical findings for consideration for improving healthcare delivery. However, there are limitations that must be considered in the interpretation of the results. Several studies have assessed malaria case-management among different populations using different approaches [13, 19–23]. In our study, we used direct observation of consultations to explore this subject using an adaptation of the LQAS technique. Strict comparison of the results is therefore challenging and must be done cautiously. Another limitation is the Hawthorne effect. Since the health workers were observed directly, they were likely to perform to impress the observers therefore leading to overestimation of case-management indicators. On the other hand, by virtue of the fact that they were observed, they could become nervous and underperform resulting in underestimation of the indicators.

## Conclusion

Malaria case-management in the KSD was satisfactory with almost universal diagnostic testing. However, inappropriate prescription of anti-malarials, complete assessment of children and counseling of patients were some of the tasks deficient. Use of ACTs however, was the standard practice in accordance with national treatment guidelines but use of AA as recommended first line drug was low in favour of alternative first line AL. Malaria control interventions targeted at the health workers are warranted. Sensitization of health workers on the malaria case-management guidelines should be intensified.
